# *MET* Gene Dysregulation as a Promising Therapeutic Target in Lung Cancer—A Review

**DOI:** 10.3390/jpm11121370

**Published:** 2021-12-14

**Authors:** Paulina Terlecka, Paweł Krawczyk, Anna Grenda, Janusz Milanowski

**Affiliations:** Department of Pneumonology, Oncology and Allergology, Medical University of Lublin, 20-090 Lublin, Poland; pawel.krawczyk@umlub.pl (P.K.); anna.grenda@umlub.pl (A.G.); janusz.milanowski@umlub.pl (J.M.)

**Keywords:** non-small cell lung cancer, *TKI MET*, amplification, skipping mutations

## Abstract

Several molecular abnormalities in the *MET* gene have been identified, including overexpression, amplification, point mutations, and “skipping mutation” in exon 14. Even though deregulated *MET* signaling occurs rarely in non-small cell lung cancer (NSCLC), it possesses tumorigenic activity. Since the discovery of the significant role played by *MET* dysregulations in resistance to epidermal growth factor receptor tyrosine kinase inhibitors (EGFR TKI), many clinical trials have been focused on mechanisms underlying this acquired resistance. Therefore, new therapeutic strategies are being considered in the personalized therapy of NSCLC patients carrying *MET* abnormalities. First, MET kinase inhibitors (tepotinib and capmatinib) have been shown to be effective in the first and subsequent lines of treatment in NSCLC patients with “skipping mutations” in exon 14 of *MET* gene. In this article, the authors show the role of MET signaling pathway alterations and describe the results of clinical trials with MET inhibitors in NSCLC patients.

## 1. Introduction

Lung cancer is the most common malignant neoplasm in the world, constituting a serious global health problem due to the very poor prognosis. As shown in the 2018 WHO (World Health Organization) report, lung cancer accounted for 11.6% of all diagnosed malignant neoplasms, which corresponds to approximately 2.1 million patients. Due to its varied characteristics, a simplified division of lung cancer into small cell (SCLC) and non-small cell lung cancer (NSCLC) is used in clinical practice. The life expectancy in NSCLC patients has increased in recent years thanks to the latest therapies. The promising results of clinical trials encourage searching for rare molecular factors that may be important in personalized therapy in NSCLC patients. The genetic disorders in neoplastic cells identified currently exert an enormous impact on the choice of the treatment method.

## 2. Testing of *MET* Gene Abnormalities

One of the mechanisms observed in malignant tumors involves impairment of signaling by MET receptor tyrosine kinase. In some neoplasms, abnormalities in the MET pathway are quite common, e.g., in hereditary papillary renal carcinoma, liver cancer, or head and neck carcinoma. However, *MET* gene abnormalities represent rare genetic changes in NSCLC patients. The registration of capmatinib and tepotinib MET inhibitors in Europe and the United States for NSCLC patients with *MET* exon 14 mutations has contributed to popularization of investigations of multiple genes, including *MET* gene, with the Next Generation Sequencing (NGS) technique using target sequencing or Comprehensive Genomic Profiling (CGP).

In some countries, the standard procedure consists of the detection of mutations in exon 14 of the *MET* gene. Such analyses should be performed when *EGFR* gene mutations, and *ALK* and *ROS1* gene rearrangements have been excluded or when the diagnosis is based on the NGS technique. The NGS technique seems to be the most promising method for the detection of “skipping mutations” of the *MET* gene, which is related to the great diversity and numerous variants of this genetic abnormality. Moreover, NGS analyses can be performed not only in material collected from the tumor or metastasis (Formalin-Fixed Paraffin-Embedded, FFPE) but also in circulating free DNA (cfDNA) or mRNA from peripheral blood (the so-called liquid biopsy). Attempts have been made to detect *MET* gene mutations with the use of reverse transcription real-time PCR (RT-PCR), which is used for transcription of mRNA into complementary DNA (cDNA). However, this technique turned out to be too insensitive and nonspecific, especially in analysis of FFPE materials [1,2].

Other genetic tests that can be performed and are not part of the routine molecular diagnostics of lung cancer include the *MET* gene amplification test, especially in patients with progression during EGFR TKI therapy in the absence of the Thr790*Met* resistance mutation in exon 20 of the *EGFR* gene. The copy number variation (CNV) of the *MET* gene can be analyzed with NGS or FISH (Fluorescence In Situ Hybridization) methods. FISH technique uses molecular fluorochrome-labeled probes and fluorescence microscopy [3].

The listed methods provide completely different results. NGS should be the gold standard in *METex14* mutations analysis conducted in tumor or liquid biopsy materials. It is a sensitive method and detects all splicing mutations variants. RT-PCR testing should no longer be used for this purpose. This technique may have insufficient sensitivity and specificity and may not detect all variants of splicing mutations. Completely different goals are targeted by IHC and FISH techniques. These techniques do not have the diagnostic power to detect *METex14* mutations. In the IHC method, high MET protein expression is defined as a high percentage of neoplastic cells with detected MET expression or as strong MET staining on neoplastic cells. Moreover, the *MET* gene copy number considered amplification of this gene has not been precisely determined. The MET protein expression determined with IHC correlates only slightly positively with the *MET* gene copy number determined by FISH. Therefore, it is difficult to say whether the study of MET protein expression or *MET* gene copy number could be a reliable predictive factor in qualifying as a treatment with MET inhibitors [3,4].

The summary of techniques used to *MET* abnormalities examination is compiled in the Table 1.

## 3. *MET* Gene Abnormalities in NSCLC Patients

**Figure 1:**
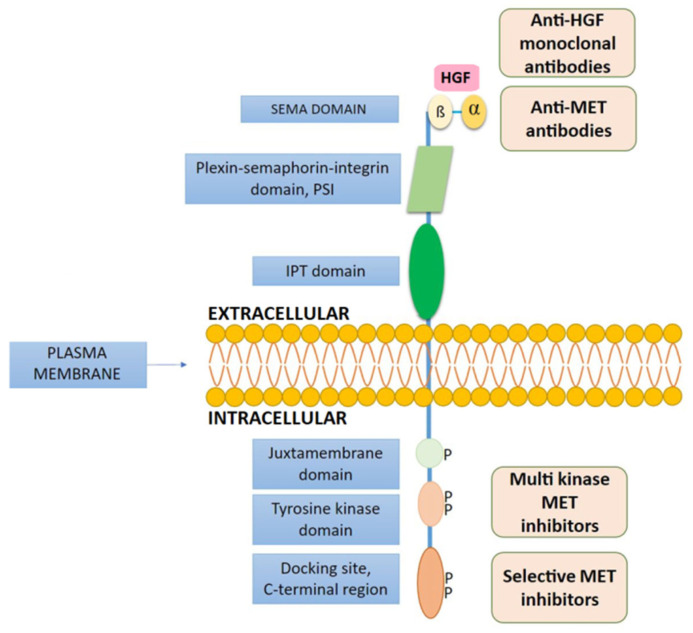
Structure of the MET protein with indication of its domains and sites of action on the MET receptor for targeted therapies. (SEM—semaphorins domain; PSI—plexin-semaphorin-integrin domain; IPT—Immunoglobulin-like fold, Plexins, Transcription factors).

The *MET* gene is located on chromosome 7 (*locus* 7q31). It consists of a sequence of 21 exons and 20 introns. It codes the c-MET protein (c-Mesenchymal-Epithelial Transition Factor)—a transmembrane receptor with tyrosine kinase activity, which is activated by the hepatocyte growth factor (HGF). Hence, c-MET is also referred as a Hepatocyte Growth Factor Receptor (HGFR) (Figure 1). HGFR is a 190 kDa glycoprotein composed of an extracellular transmembrane domain (TM) and an intracellular domain. The intracellular portion contains a juxtamembrane domain (JX), a tyrosine kinase domain (TK), and a C-terminal part, which is the site of signal and adapter protein interactions. The N-terminal extracellular part consists of a semaphorin domain responsible for HGF binding and a cysteine-rich site [5]. HGF, also referred to as the scatter factor (SF), represents the plasminogen family but has no enzymatic activity and is produced by various cells, e.g., epithelial cells, endothelial cells, neurons, hepatocytes, and hematopoietic cells [6,7,8].

The activation of HGFR leads to dimerization and autophosphorylation of tyrosine residues in the C-terminal kinase domain. The enhancement of c-MET kinase activity through activating mutations or amplification of the *MET* gene, overexpression of HGFR, or overproduction of HGF results in the recruitment of adapter and signal proteins and further activation of intracellular pathways and signaling via the RAS-MAPK and PI3K-AKT pathways to the cell nucleus [9]. This results in increased multiplication of neoplastic cells, invasion, angiogenesis, and metastasis [10].

To date, HGFR abnormalities have been mainly associated with the development of papillary renal cell carcinoma. However, impaired signaling of HGFR tyrosine kinase caused by its overexpression or excessive activation has been evidenced in the pathogenesis of other neoplasms, including NSCLC [11]. MET pathway activation has clinical significance, either by primary oncogenic driver mutations or in the mechanism of development of acquired resistance to EGFR TKIs.

In NSCLC patients, the most important factors in the choice of therapy are splicing mutations (deletions, insertions, and substitutions) in introns 13 (splice donor) and 14 (splice acceptor) and in exon 14 of the *MET* gene (“skipping mutations”, *METex14*). The splice site mutation exerts an impact on post-transcriptional processing during mRNA splicing for the *MET* gene when exon 14 is skipped [12]. The molecular mechanism leading to deregulation of the c-MET function is related to the loss of the ligase binding site (Casitas B-lineage Lymphoma, CBL), which is involved in the ubiquitination of proteins destined for degradation. The CBL binding site in the JX region of HGFR is encoded by exon 14, which loses its function due to the splicing mutation. In normal conditions, CBL binds to MET, allowing for ubiquitination and subsequent degradation of tyrosine kinase by lysosomes. During normal signaling, this process allows the receptor to be shed in a controlled manner from the cell surface. The absence of CBL binding prolongs the action of the receptor (the receptor retains its affinity for HGF), increases the density of the receptor on cells, and leads to excessive intracellular signaling [11,12,13,14,15,16,17,18,19,20].

Splicing mutations are mainly observed in patients with lung adenocarcinoma and in 3–4% of patients with this type of NSCLC [21]. *METex14* may occur in 1% of patients with squamous cell lung cancer and only in 0–0.2% of patients with SCLC [22]. *METex14* is observed more frequently in women than in men, and the age of patients with this genetic abnormality is usually between 70 and 75 years.

The amplification and skipping mutations in exon 14 of the *MET* gene are an unfavorable prognostic factor in NSCLC patients (greater tendency toward development of distant metastases). Nevertheless, they facilitate establishment of targets for molecularly targeted therapies [23,24,25]. Neoplastic cells with *METex14* are mostly sensitive to the MET tyrosine kinase inhibitors (MET TKI) treatment. Therefore, this abnormality has been classified as the so-called driver mutation in NSCLC patients.

Small molecule MET TKI can be divided into three types (I, II, and III) depending on the mode of binding and inhibition of catalytic activity. Type I includes molecules that are bound to the ATP-pocket in the active form of MET (Asp-Phe-Gly motif with “in” conformation, DFG-in). In turn, type II inhibitors bind the ATP-pocket in the inactive state (Asp-Phe-Gly motif with “out” conformation, DFG-out). Both types I and II are ATP-competitive HGFR kinase inhibitors [22,26], which include cabozantinib (VEGFR2, MET, AXL, TIE2, FLT3, ROS1, KIT, and RET inhibitors), capmatinib (the MET inhibitor), tepotinib (the MET inhibitor), savolitinib (the MET inhibitor), crizotinib (ALK, ROS1, and MET inhibitors), foretinib (MET and VEGFR2/KDR inhibitors), merestinib (ROS1, AXL, RON, MERTK, FLT3, DDR1/2, MST1R MKNK-1/2, and MET inhibitors), glesatinib (MET and AXL inhibitors), sitrawatinib (MET, AXL, MERTK, VEGFR, PDGFR, KIT, FLT3, TRK, RET, DDR2, and EP inhibitors), SU 11274, PHA-665725, MK-2461, AMG- 458, AMG- 337, and PF-04217903. Finally, tivantinib is a type III non-ATP-competitive HGFR kinase inhibitor (Figure 1) [27].

In this review, we focused on therapeutic options not only in patients with exon 14 mutations in *MET* gene but also in patients with *MET* gene amplification and MET protein overexpression. This approach is rare in this type of study.

## 4. *HGFR* Inhibitors in Treatment of Patients with EGFR TKI Resistance and *MET* Gene Amplification

*MET* gene amplification is rarely observed in untreated patients with advanced NSCLC (2–4% of patients). It may occur slightly more frequently in patients with *EGFR* gene mutations, which may induce primary resistance where a cancer does not respond to EGFR TKI applied as primary therapy strategy (5% of patients). Its appearance may induce the acquired resistance of tumor cells to EGFR TKI in approximately 20% of EGFR TKI-treated patients [28,29,30,31,32,33]. Acquired resistance means cancer initially responded to therapy but, after a period of time, it relapsed and progressed. HGFR overexpression caused by *MET* gene amplification activates alternative intracellular pathways, including HER3 and the HER3-dependent PI3K-AKT pathway, which is independent of the presence of the Thr790*Met* mutation in exon 20 of the *EGFR* gene [34,35].

Patients with *MET* amplification and resistance to EGFR TKI may benefit from therapy based on MET TKI. In clinical trials, attempts are being made to find molecules that could overcome the resistance mechanism or to prolong the response to EGFR TKI therapy. One of such attempts was the therapy based on an anti-MET antibodies, i.e., onartuzumab (METLung trial) or a small molecule MET inhibitor tivanitinib (MARQUEE trial) in combination with erlotinib (first-generation EGFR TKI) in the second and subsequent lines of treatment. The randomized phase III METLung trial evaluated the efficacy of a combination of onartuzumab with erlotinib or erlotinib monotherapy in second- or third-line treatments in c-*MET* positive patients with stage IIIB or IV NSCLC (≥50% of cancer cells with 2+ or 3+ HGFR expression in IHC evaluation). The therapy was applied in 499 patients with NSCLC with varied *EGFR* gene status. Onartuzumab in combination with erlotinib did not improve survival rates and did not prolong progression-free survival (PFS), and the median of overall survival (OS) in patients receiving onartuzumab with erlotinib was lower than in patients treated with erlotinib alone (6.8 versus 9.1 months, respectively; HR = 1.27; 95% CI: 0.98–1.65; *p* = 0.067) [36].

The phase III MARQUEE trial assessed the efficacy of combined therapy with tivanitinib and erlotinib, and erlotinib monotherapy in the second- or third-line treatments in patients with non-squamous NSCLC in stage IIIB or IV, depending on the expression of c-*MET*. The study found that, compared with the erlotinib monotherapy, the combination of tivanitinib and erlotinib treatment increased the median PFS (3.6 versus 1.9 months; HR = 0.74; 95% CI: 0.62–0.89; *p* = 0.001), but the differences in the OS were not statistically significant (8.5 versus 7.8 months, HR = 0.98; 95% CI: 0.84–1.15; *p* = 0.81). The combination therapy was found to be more beneficial than the erlotinib monotherapy only in patients with high (copy number 4 or higher) *MET* gene amplification (median OS: 9.3 versus 5.9 months, HR = 0.70; 95% CI: 0.49–1.01). In turn, in patients with EGFR gene mutations, who constituted only 10.4% of the study group, a significant increase in the median PFS and OS was observed after the application of the combination therapy than erlotinib alone. The median PFS was 13.0 months in patients receiving erlotinib with tivanitinib and 7.5 months in patients treated with erlotinib alone (HR = 0.49; 95% CI: 0.31–0.77). The median OS was 25.5 months and 20.3 months, respectively (HR = 0.68; 95% CI: 0.43–1.08) [37].

Regarding the two trials presented above, it should be emphasized that no *EGFR* gene activating mutation in cancer cells was detected in a vast majority of the enrolled patients. Therefore, it was expected that the efficacy of EGFR TKI-based combination therapies would be unsatisfactory in this group of patients [38]. Consequently, the combination second- or third-line therapies with MET TKI and EGFR TKI in non-squamous NSCLC patients in stage IIIB or IV were not approved.

Patients with a mutation in the *EGFR* gene participated in a further study. Promising results were provided by a phase Ib/II trial, in which capmatinib was applied in combination with gefitinib in patients with EGFR TKI resistance and HGFR overexpression or *MET* gene amplification. In total, 161 patients were enrolled in the study, and 27% of all patients received capmatinib and gefitinib in phase Ib and II trials. The most beneficial effect of such a therapy was achieved in patients with *MET* amplification described as a ≥6 gene copies in cancer cells. The overall response rate (ORR) in such a group of patients was 47% [39].

In the phase Ib TATTON trial, an attempt was made to overcome resistance to EGFR TKI of all generations in a combination therapy with savolitinib (MET TKI) and osimertinib (third-generation EGFR TKI) in NSCLC patients with EGFR gene mutations and *MET* gene amplification. Response to the combination therapy was reported in 33% of patients previously treated with osimertinib, 55% of patients with the *Thr790Met* mutation previously treated with first- or second-generation EGFR TKI, and 61% of patients without the *Thr790Met* mutation previously treated with erlotinib, gefitinib, or afatinib [40]. In 2019, further results of an assessment of the effectiveness of the aforementioned combination in patients with *EGFR* gene mutations and resistance to EGFR TKI as well as *MET* gene amplification were presented during the American Association for Cancer Research (AACR) Annual Meeting. In patients with resistance to first- and second-generation EGFR TKI (regardless of the resistance mechanism), the value of ORR was 52% and the median Duration of Response (DoR) was 7.1 months. In turn, in the group of patients receiving savolitinib to overcome resistance to osimertinib, the ORR value was only 25% but the median DoR was 9.7 months. Unfortunately, the combination therapy was characterized by relatively high toxicity and caused nausea (67% of patients), rash (56% of patients), and vomiting (50% of patients) [41].

Clinical trials are underway to assess the bispecific anti-MET and anti-EGFR antibody, i.e., amivantamab, used in combination with third-generation EGFR TKI lazertinib in osimertinib-resistant NSCLC patients. A phase I study evaluated the efficacy and safety of amivantamab in patients with advanced NSCLC harboring *EGFR* mutations. A total of 116 NSCLC patients with *EGFR* mutations were enrolled in the study, and 28% of patients achieved partial remission. In 47 patients with resistance to third-generation EGFR TKI, 21.3% patients obtained partial remission, including four patients with C797S substitution in *EGFR* gene, one patient with *MET* amplification, and five patients without *EGFR* and *MET* genes alterations. In 20 patients with exon 20 mutations in *EGFR* gene, 30% achieved partial response [42].

Clinical trials were also undertaken to assess the application of anti-HGF monoclonal antibodies (rilotumab, ficlatuzumab, and TAK 701) that bind to HGF, preventing its binding to HGFR and inhibiting the c-MET signaling pathway. Rilotumumab is a fully humanized IgG2 anti-HGF monoclonal antibody. A phase 1/2 study evaluated the efficacy and safety of rilotumumab in combination with erlotinib in NSCLC patients regardless of the *EGFR* gene status. The overall response rate was only 8%; however, the disease control rate was 60%. The median PFS was 2.6 months (90% CI: 1.4–3.3 months), and the median OS was 6.6 months (90% CI: 5.6–8.9 months). Ficlatuzumab is a humanized IgG1 monoclonal antibody anti-HGF. A randomized phase II clinical trial evaluated the efficacy of gefitinib with or without ficlatuzumab in patients with NSCLC. In the group with *EGFR* mutations and high expression of MET on cancer cells, 41% of patients treated with ficlatuzumab and gefitinib had response to therapy compared with 22% of patients with response to gefitinib therapy. The median PFS was 11 versus 5.5 months, respectively. However, in the whole population, the combination therapy compared with the gefitinib monotherapy did not significantly improve the overall response rate (40% versus 38%), PFS (5.6 versus 4.7 months), and OS (24.7 versus 21.8 months). Ficlatuzumab plus gefitinib can improve the clinical efficacy in NSCLC patients with EGFR mutations and low c-MET expression, indicating that MET expression may be a biomarker for ficlatuzumab treatment [43,44,45,46].

The information on clinical trials where MET inhibitors were used in patients with acquired resistance to EGFR TKI is compiled in Table 2.

*MET* gene amplification also occurs in EGFR TKI-untreated NSCLC patients. In the first-phase study PROFILE 1001 (NCT00585195), it was found that, in previously treated patients with amplification of the *MET* gene described as ≥5 gene copies, the ORR to crizotinib therapy was 40% (95% CI: 19.1–63.9) and the median PFS was 6.7 months (95 CI: 3.4–7.4), while in the group of patients with low amplification of the *MET* gene (≥2–<5 gene copies), ORR was 14.3% with the same median PFS [47]. In the AcSe study, 25 patients with *MET* gene amplification received crizotinib. Response to treatment was reported in 32% of patients, and the median PFS and OS were 3.4 months and 7.7 months, respectively [48]. At present, a phase II NCT03539536 trial is underway to evaluate the safety and efficacy of application of telisotuzumab vedotin (teliso-v)—an anti-MET antibody conjugated with MMAE (Monomethyl Auristatin E)—a spindle tubulin inhibitor. Teliso-v is used in second- or third-line therapies in patients with stage IIIB or IV NSCLC and overexpression of c-*MET*. The phase I trial (NCT02099058) evidenced the safety and beneficial anti-tumor effects of teliso-v [49].

## 5. Treatment of Patients with *MET*ex14 Mutations

Clinical trials have shown that crizotinib, capmatinib, glesatinib, AMG337, and tepotinib may be effective in the treatment of NSCLC patients with a confirmed *METex14* mutation. In 2015, the descriptions of four patients with stage IV NSCLC adenocarcinoma and a *METex14* mutation who responded to therapy with cabozantinib or crizotinib after 4, 6, or 8 weeks of therapy were presented for the first time. Complete PET response according to PERCIST (PET Response Criteria in Solid Tumors) was observed in the liver of a patient treated with cabozantinib, and partial responses according to RECIST (Response Evaluation Criteria in Solid Tumors) in lungs were reported in the other three patients treated with crizotinib. It was one of the most important reports on the possibility of effective treatment with cabozantinib or crizotinib in lung adenocarcinoma patients with a “skipping mutations” of exon 14 of the *MET* gene. Since half of the patients did not have accompanying *MET* amplification, it was confirmed that the response to the therapy in these patients was related to the presence of a splicing site mutation in exon 14 of the *MET* gene [18].

The efficacy of MET TKI in patients with lung adenocarcinoma with the *METex14* mutations was the subject of a multicenter retrospective clinical trial, which included 148 patients. Among 61 patients with stage IV NSCLC, 27 were qualified for therapy with one of the MET TKI, and the others were the control group. The majority of patients receiving MET TKI were treated with off-label crizotinib (20 patients). Four patients received this drug as a part of the clinical trial, and the others received glesatinib (four patients) or capmatinib (four patients). In patients treated with crizotinib, the median PFS was 7.36 months and the median OS was 20.5 months (95% CI: 9.5–NR). In all patients treated with MET TKI, the median OS was 24.6 months (95% CI: 12.1–NR). In turn, in patients who did not receive MET TKI, the median OS was 8.1 months (95% CI: 5.3–NR). The prognosis in patients receiving the standard of care was worse in the group with *MET* gene amplification (5.2 versus 10.5 months, *p* = 0.06). As revealed by this analysis, treatment with MET TKI is associated with improvement in OS of patients with advanced lung adenocarcinoma and a confirmed *METex14* mutation [50].

In 2018, updated data on the response to crizotinib treatment in lung adenocarcinoma patients with *METex14* mutations were presented at the World Conference on Lung Cancer (WCLC). The patients received 250 mg of crizotinib twice daily in the PROFILE1001 trial. The response to the therapy was assessed using the RECIST v 1.0 criteria. The study group comprised 69 patients, mostly with lung adenocarcinoma (84%). The ORR to the crizotinib treatment was 32% (95% CI: 21–45). Three patients reached complete response (CR), eighteen patients achieved partial response (PR), and twenty-nine patients achieved stable disease (SD). The median PFS was 7.3 months (95% CI: 5.4–9.1) [1].

The efficacy of crizotinib in chemotherapy-treated stage IV NSCLC patients with abnormalities in the *MET* gene (amplification or mutations) was the subject of an AcSe study conducted in 2013–2018 (Table 2). The drug was administered orally at a dose of 250 mg twice a day. The response to the treatment was assessed after two RECIST 1.1 cycles with the use of computed tomography or magnetic resonance imaging. The secondary efficacy assessment included the best overall response rate, PFS, and OS. The efficacy of crizotinib was demonstrated in patients with confirmed various *MET* gene mutations (28 patients in total). The most beneficial effect was recorded in a group of patients with the *METex14* mutation. The group of 25 patients was characterized by the highest ORR (40%), median PFS of 3.6 months (95% CI: 1.6–7), and median OS of 9.5 months (95% CI: 4.1–13.4) [48].

Based on the conclusions of the PROFILE1001 and AcSe studies, crizotinib was the first MET TKI to receive a Breakthrough Therapy designation in May 2018 from the FDA (U.S. Food and Drug Administration) for the treatment of patients with metastatic NSCLC previously treated with platinum-based chemotherapy and with *MET* exon 14 alterations [12].

## 6. Other MET Inhibitors in the Treatment of NSCLC Patients with *METex14* Mutations and other *MET* Gene Abnormalities

### 6.1. Capmatinib

Capmatinib (INC280) is a highly potent and selective inhibitor of the MET receptor. In addition, capmatinib crosses the blood–brain barrier. Preliminary clinical data showed low-grade toxic effects and promising efficacy of capmatinib monotherapy in NSCLC patients with *MET* gene dysregulation. Phase I clinical trial enrolled 55 advanced NSCLC patients with *MET* gene abnormalities, and 73% of the patients received two or more prior systemic therapies. The ORR was 20% (95% CI: 10.4–33.0). In the group of patients with ≥6 *MET* gene copy number, 47% achieved partial response and median PFS of 9.3 months (95% CI: 3.8–11.9). Tumor responses were observed in all four patients with the *MET*ex14 mutation. The most common toxicities were nausea (42%), peripheral edema (33%), and vomiting (31%) with a low degree of severity [51].

On 6 May 2020, capmatinib was approved by the FDA for the treatment of stage IV NSCLC patients with a confirmed *MET*ex14 mutation [24]. This drug is expected to be registered in the European Union in 2022. The registration was based on the results of a multicenter, non-randomized, multi-cohort phase II GEOMETRY mono-1 trial, which assessed the efficacy and safety of capmatinib in 364 advanced NSCLC patients with a *MET*ex14 mutation or *MET* gene amplification.

In this study, the oral administration of capmatinib at a dose of 400 mg twice daily resulted in a high response rate, especially in first-line treatment. Among NSCLC patients with *MET* exon 14 “skipping mutation”, the ORR was 41% (95% CI: 29–53) in a group of patients who had received one or two lines of therapy and 68% (95% CI: 48–84) in a group of patients who had not received prior treatment. The median duration of the response was 9.7 months (95% CI: 5.6–13.0) and 12.6 months (95% CI: 5.6–NE), respectively. The median PFS was 5.4 months (95% CI, 4.17–6.97) in previously systemically treated patients and 12.4 months (95% CI: 8.2–NE) in untreated subjects. Limited efficacy of capmatinib was observed in previously treated patients with *MET* amplification who had less than 10 gene copy number (overall response rate: 9%, 95% CI: 7–12). Among patients with *MET* amplification and ≥10 gene copy number, response was observed in 29% (95% CI: 19–41) of previously treated patients and in 40% (95% CI: 16–68) of patients who had not received treatment previously. The median DoR in these patients was 8.3 months and 7.5 months, and the median PFS was 4.1 months and 4.2 months, respectively. The most frequently reported adverse events of capmatinib therapy were peripheral edema (in 51% of patients) and nausea (in 45% of patients). However, these events were mostly of grade 1 or 2 [52].

A current clinical trial aimed to compare the efficacy of capmatinib in NSCLC patients with the *MET*ex14 mutation to the efficacy of docetaxel in second-line treatment (GEOMETRY-III). As established in preclinical studies, capmatinib in combination with anti-PD-1 (Programmed Death 1) antibodies has immunomodulatory effects regardless of the presence of genetic changes in the *MET* gene. Currently, numerous phase I and II clinical trials are underway (Table 2), in which various regimens are used in combination with capmatinib and anti-PD-1 agents (pembrolizumab and spartalizumab), chemotherapy, or EGFR TKI (osimertinib) in locally advanced or metastatic NSCLC patients with or without the presence of *MET* gene dysregulation (GEOMETRY-C, GEOMETRY-E, NCT04139317, NCT04323436, NCT03333343, and STARTER_cMET).

### 6.2. Tepotinib

Tepotinib is a once-daily highly selective oral MET inhibitor that has shown promising clinical activity in cancer patients with *MET* abnormalities. In a phase I clinical trial (NCT01014936), 149 patients with solid tumor received different doses of tepotinib. The dose of tepotinib was established at 500 mg once daily. The treatment-related adverse events mostly included grades 1 or 2 fatigue, peripheral edema, decreased appetite, nausea, vomiting, and lipase increase. The best overall response was the partial response in two patients with *MET* overexpression. Only two NSCLC patients with HGFR overexpression and *MET* gene amplification but without *METex14* mutations participated in this study [53].

The efficacy of tepotinib administered orally once daily at a dose of 500 mg in 152 lung adenocarcinoma patients with a mutation exon 14 “skipping mutations” of the *MET* gene (detected with the use of NGS in cfDNA or in the tumor) was demonstrated in a multicentre phase II VISION trial. The ORR was 46% (95% CI: 36–57), with a median duration of the response of 11.1 months (95% CI: 7.2–NE), and 48% patients with a positive molecular result in cfDNA (*n* = 66) achieved complete or partial remission. The ORR was 50% in 60 patients with *METex14* mutation detected in tissue biopsy, and 27 patients had positive results shown by both methods. A similar response rate was observed regardless of the treatment line and in patients with locally advanced and metastatic NSCLC. A molecular response measured in circulating free DNA was observed in 67% of the patients with matched liquid-biopsy samples at the baseline and during treatment. Adverse events of grade 3 or higher to tepotinib therapy were reported in 28% of the patients, including peripheral edema in 7% of the patients. The adverse events led to discontinuation of tepotinib in 11% of the patients [18,54].

The study showed the activity of tepotinib in NSCLC patients with *METex14* mutations. Given the results, on 3 February 2021, the FDA accelerated the approval for application of tepotinib in metastatic NSCLC patients with the *METex14* mutation. Nevertheless, further approval decisions will depend on the clinical benefits of tepotinib therapy assessed in subsequent clinical trials.

### 6.3. Cabozantinib

Cabozantinib is an inhibitor of VEGFR2, MET, AXL, and RET. Cabozantinib was the first, orally available MET inhibitor to enter clinical trials in 2005. Currently, cabozantinib has been approved by the FDA for treatment of metastatic medullary thyroid cancer and first-line treatment of advanced renal cell carcinoma and hepatocellular carcinoma patients previously treated with sorafenib.

In ongoing studies assessing the use of MET TKI in NSCLC patients with the *MET*ex14 mutation, cabozantinib exhibited activity against central nervous system (CNS) metastases. The first report was based on the report of a patient qualified for the phase I PROFILE1001 trial. Due to the metastatic progression into the CNS, crizotinib was replaced by cabozantinib and a reduction in metastatic lesions was observed [55].

Although a large group of NSCLC patients with the *METex14* mutation is treated with cabozantinib in clinical trials, their results have not been published yet. However, several case reports have demonstrated the safety and potential activity of cabozantinib in NSCLC patients with the *METex14* mutation. An Italian phase II trial is currently evaluating cabozantinib in NSCLC patients with *MET* gene amplification or *METex14* mutation (NCT03911193).

### 6.4. Glesatinib

Glesatinib is a multi-targeted inhibitor with affinity to c-MET; TEK/TIE-2; RON; SMO; and VEGFR types 1, 2, and 3. In one clinical case report, a NSCLC patient with the *METex14* mutations showed response to glesatinib after relapsing to crizotinib, including remission of liver metastases. The AMETHYST NSCLC trial is a global phase II study enrolling NSCLC patients after systemic treatment with *MET* gene alterations detected in tumor tissue or in cfDNA. It was shown that, in patients harboring *MET* gene activating mutations in tumor tissue (*n* = 28), the ORR was 10.7% (95% CI: 2.27–28.23) and the median PFS was 3.95 (95% CI: 2.11–4.18). In turn, response was observed in 25% (95% CI: 3.19–65.09) of patients with *MET* gene mutations detected in cfDNA (*n* = 8). In this group of patients, the median PFS was 3.39 (95% CI: 1.28–NE). The 1-year survival rates were 50.47% (95% CI: 27.49–69.62) and 54.69% (95% CI: 13.72–83.24) [22].

### 6.5. Bozitinib

Bozitinib (APL-101) is a highly selective and specific MET inhibitor. NCT03175224 is a phase I/II international multicenter open-label study evaluating the safety, pharmacokinetics, and preliminary efficacy of bozitinib in NSCLC patients with the *METex14* mutation. Based on completion of the phase I and approval from the safety review committee to advance the trial, the phase II clinical trial SPARTA was initiated. Another phase II study (NCT04258033) has recently been initiated in China and will include 185 advanced NSCLC patients harboring *MET* dysregulation to assess the efficiency and safety of bozitinib [22].

### 6.6. Anti-MET Monoclonal Antibodies and Immunotherapy in NSCLC Patients with MET Abnormalities

The efficacy of onartuzumab—the first anti-MET antibody—was described in the section on the treatment of patients with resistance to EGFR TKI. We also mentioned a clinical trial with amivantanab—bisepcific antibody anti-MET and anti-EGFR used in this indication. Two anti-HGF antibodies (rilotumab and ficlatuzumab) used in combination with erlotinib or gefitinib to increase the efficacy of EGFR TKI have also been described. However, there are other monoclonal antibodies that have been used in clinical trials for NSCLC patients with *MET* abnormalities.

Emibetuzumab is anti-MET monoclonal antibody that blocks the binding of HGF, leading to internalization and degradation of MET. A phase II clinical trial (NCT01900652) evaluated the efficacy and safety of emibetuzumab in monotherapy or in combination with erlotinib in NSCLC patients with MET expression. Regardless of the treatment method, response to treatment occurred in less than 5% of patients, and the median PFS, even in patients with high MET expression, was 3.3 months in the group receiving the combined treatment and 1.6 months in group treated only with emibetuzumab. Another phase II clinical trial (NCT01897480) evaluated the efficacy and safety of erlotinib with or without emibetuzumab in patients with advanced NSCLC harboring *EGFR* mutations. There were no differences in PFS depending on the treatment method in the overall population. However, in patients with MET high expression, the combination treatment significantly prolonged PFS compared with erlotinib monotherapy (20.7 versus 5.4 months; HR = 0.39; 90% CI: 0.17–0.91).

Telisotuzumab is a novel anti-MET antibody conjugated with monomethyl *staphylococin E* (MMAE), which mediates cancer cells apoptosis. In a phase I study, 18.5% of patients with high expression of MET obtained partial remission. The median response duration was 4.8 months, and the median PFS was 5.7 months

Changes in MET expression leads to pathological consequences: tumorigenesis, cancer progression, mediation of anti-cancer drug resistance, or regulation of immune response, mainly modulating dendritic cells functions. The MET signaling pathway is involved in the immune response, e.g., required for chemoattraction and neutrophil-mediated cytotoxicity. MET also affects APCs (Antigen Presenting Cells), increases their functions, and activates regulatory T-cell (CD4+), thus eventually control cytotoxic T-cells (CD8+), playing a positive role in anti-cancer immunity. On the other hand, MET may act as a negative regulator of APCs, leading to an increase in immunosuppressive factors such interleukin-10 (IL-10) and transforming growth factor beta (TGF-β) [56].

There are only a few scientific reports assessing the effectiveness of the combination of immunotherapy and MET inhibitors in patients with NSCLC. Further clinal evaluation are needed suggesting that combining MET inhibition with anti-PD1 (Programmed Death 1) or anti-PD-L1 (Programmed Death Ligand 1) treatment has a role in increasing immunotherapy efficacy.

In 2021, Kron et al. assessed the type of *MET* aberration, co-occurring mutations, and PD-L1 expression using NGS, in situ hybridization techniques and immunohistochemistry. These researchers concluded that patients with *METex14* mutation do not seem to benefit from immunotherapy in contrast with patients with *MET* gene amplified tumors. This is especially important for the poor prognosis subgroup with the overexpression of MET and with *MET* gene copy number ≥ 10 [57].

Despite such opinions, there are several clinical trials of combination therapy trials involving MET inhibitors and immune check points inhibitors. One of them is the combination of treatment with capmatinib and spartalizumab (anti-PD-1 monoclonal antibody). The first clinical trial conducted in 18 NSCLC patients, regardless of *MET* gene status, ended negatively. However, a study with a combination of these drugs is ongoing in NSCLC patients with a *METex14* mutation. Sun and co-authors identified a potential bispecific monoclonal antibody, targeting both MET and PD-1 proteins. Their study showed inhibition towards MET-mediated proliferation, migration, and stimulation cancer cells apoptosis. Such treatment also promoted T cell activation [58,59].

## 7. Conclusions

In summary, the analysis of *MET* gene abnormalities in patients with locally advanced or advanced NSCLC should be a routine diagnosis in subjects without other predictive factors such as *ROS1* or *ALK* gene rearrangements and *EGFR* gene mutations. Thanks to new generations of drugs, patients with *MET* dysregulations have a chance to substantially extend their life expectancy. The most important abnormality that qualifies as molecularly targeted therapies is skipping mutations of exon 14 in the *MET* gene. Even today, when this genetic abnormality is detected in patients with advanced NSCLC, it is possible to use effective therapy with capmatinib or tepotinib. However, there is a need to conduct further studies on the safety and efficacy of MET TKI in patients who have failed EGFR TKI therapy and in whom one of the main causes of resistance is the selection of a cancer cell clone with *MET* gene abnormalities.

## Figures and Tables

**Table 1:** Summary technique used to *MET* abnormalities examination.

Detection Method	Technique	Detected *MET* Disorder	Material for Testing	Advantages	Disadvantages
NGS	Identification of the nucleotide sequence in the targeted regions/genes including *MET* (targeted sequencing); identification of substitutions, insertions/deletions, CNV, and rearrangements/fusions in a one test (CGP)	*METex14* mutations, and other mutations, CNV, including amplification, rearrangements/fusions	DNA and RNA isolated from FFPE, cfDNA	Sensitive method with the possibility of DNA and RNA evaluation together	High costs of reagents, low availability of sequencers in laboratories; long samples preparation procedure for essential sequencing; the need for time-consuming bioinformatics analysis
qRT-PCR	Identification of mRNA with *MET* skipping mutation using molecular probes used for qPCR reactions, with prior rewriting of RNA sequences into cDNA	*METex14*, *MET* overexpression on RNA level	RNA isolated from FFPE material	Relatively simple and cheap method	qRT-PCR testing technique have insufficient sensitivity and specificity, may not detect all of splicing mutations; risk of RNA degradation, which requires special attention during preparation
FISH	Molecular fluorochrome-labeled probes attaching to the DNA in the cancer nucleidetected in fluorescence microscopy	CNV including amplification	FFPE cut on a microtome and placed on microscope slides	Identification of CNV directly in the cancer nuclei	Unable to identify the *MET* skipping mutation, and point mutation; fluorescence microscope is required
IHC	Detection of MET protein expression—visualization of the antigen-antibody complex and enzyme reaction, which is then viewed under light microscopy	MET protein expression	FFPE cut on a microtome and placed on microscope slides	IHC is a widely used method in diagnostic laboratories, its availability is high	Assessment of protein expression only, without the possibility of assessing the occurrence of the *MET* skipping mutation or CNV, including the amplification of the *MET* gene or overexpression on RNA level

NGS—Next Generation Sequencing, CNV—Copy Number Variation, CGP—Comprehensive Genomic Profiling, FFPE—Formalin-Fixed-Paraffin-Embedded Tissue, cfDNA—circulating-free DNA, qRT-PCR—quantitative Reverse Transcription real-time PCR, cDNA—complementary DNA, FISH—Fluorescence In Situ Hybridization, IHC-Immunohistochemistry.

**Table 2:** Clinical trials investigating MET TKI efficacy in NSCLC patients.

Clinical Trial Identifier	Treatment Method	Stage of NSCLC	Phase	Estimated Enrollment	Status	MET Protein and *MET* Gene Diagnostics Strategy
NCT01456325 (METlung)	Onartuzumab + erlotinib vs. erlotinib + placebo	IIIB or IV	III	499	Completed	MET expression tested by IHC
NCT01244191 (MARQUEE)	Tivantinib + erlotinib vs. erlotinib + placebo	IIIB or IV	III	1048	Terminated	MET expression tested by IHC and *MET* GCN (gene copy number) tested by FISH
NCT01887886	Erlotinib + onartuzumab vs. erlotinib + placebo	IIIB or IV	III	10	Completed	MET expression tested by IHC
NCT02031744	Erlotinib + placebo vs. erlotinib + onartuzumab	IIIB/IV	III	530	Completed	MET expression tested by IHC
NCT04427072 (GEOMETRY-III)	Capmatinib vs. docetaxel	IIIB/IIIC or IV	III	90	Recruiting	*METex14* mutation tested by NGS
NCT04816214 (GEOMETRY-E)	Capmatinib + osimertinib vs. chemotherapy (pemetrexed + cisplatin/ carboplatin)	IIIB/IIIC	III	245	Not yet recruiting	*MET* amplification measured in circulating tumor DNA (ctDNA) by real-time technique
NCT04677595 (GeoMETry-C)	Capmatinib	IIIB/IIIC or IV	II	35	Not yet recruiting	*METex14* mutation assessed in circulating tumor DNA (ctDNA) by NGS
NCT04398940	TQ-B3139	IV	II	71	Recruiting	Differecnt tests for *MET* gene abnormalities
NCT03693339 (STARTER_cMET)	Capmatinib	IV	II	27	Recruiting	*METex14* mutations tested by NGS and RT-PCR
NCT02099058	Telisotuzumab vedotin + osimertinib vs. telisotuzumab vedotin + nivolumab vs. monotherapy telisotuzumab vedotin vs. telisotuzumab vedotin + erlotinib	IV (advance solid tumors)	I	225	Recruiting	MET expression tested by IHC
NCT03539536 (2018-001772-38)	Telisotuzumab vedotin	IIIB/IV	II	310	Recruiting	MET expression tested by IHC
NCT03993873 (TPX-0022-01)	TPX-0022	IV	I	120	Recruiting	Genetic *MET* alterations including METex14 mutations, amplification, fusion or activating kinase mutation determined by NGS, FISH, quantitative polymerase chain reaction (qPCR)
NCT01639508 (12-097)	Cabozantinib	IV	II	68	Recruiting	MET overexpression, *MET* amplication or mutatation determined with different techniques
NCT02864992 (VISION)	Tepotinib	IIIB/IV	II	330	Recruiting	*METex14* mutations in plasma and/or tissue determiend by NGS
NCT04084717 (CROME/ WI235747)	Crizotinib	IV	II	50	Recruiting	*MET* activating mutation (including *METex14*) or *MET* amplification tested in plasma or tissue with different technique including NGS
NCT03940703 (2019-001538-33)	Tepotinib + osimertinib vs. tepotinib	IIIB/IV	II	120	Recruiting	*MET* amplification determined by FISH and blood-based NGS
NCT04292119 (19-629)	Lorlatinib + crizotinib vs. lorlatinib + binimetinib vs. lorlatinib + TNO155	IIIB/IV	I/II	96	Recruiting	Lack of *MET* testing, detection of *ALK* and *ROS1* rearrangement
NCT01610336	Capmatinib + gefitinib	-	II	161	Completed	
NCT04139317 (CINC280I12201)	Capmatinib (INC280) vs. pembrolizumab	IIIB/IV	II	96	Recruiting	*MET* gene copy number tested by FISH or MET overexpression tested by IHC
NCT04323436 (CINC280J12201)	Capmatinib (INC280) + spartalizumab (PDR001) vs. capmatinib + placebo	IIIB/IV	II	270	Recruiting	*METex14* mutations tested by NGS
NCT03333343 (CEGF816X2102)	EGF816 + INC280	IIIB/IV	I	157	Recruiting	-
NCT04606771	Osimertinib + savolitinib vs. savolitinib + placebo	IIIB/IV	II	56	Recruiting	*MET* amplification tested by FISH
NCT03778229 (SAVANNAH)	Osimertinib + savolitinib	IIIB/IV	II	259	Recruiting	*MET* amplifiecation and MET overexpresion tested by FISH or IHC
NCT03944772 (ORCHARD)	Osimertinib + savolitinib vs. osimertinib + gefitinib vs. osimertinib + necitumumab vs. carboplatin + pemetrexed + durvalumab vs. observational cohort—no study drug vs. osimertinib + alectinib vs. osimertinib + selpercatinib	IIIB/IV	II	150	Recruiting	*-*
NCT02954991	Glesatinib + nivolumab vs. sitravatinib + nivolumab vs. mocetinostat + nivolumab	IIIB/IV	II	206	Active, not recruiting	-
NCT03906071 (SAPPHIRE)	Nivolumab + sitravatinib vs. docetaxel	IV	III	532	Recruiting	Testing for *EGFR* mutations, *ROS1* fusions, *ALK* mutations or *ALK* fusions, *MET* not tested
NCT02664935 (ISRCTN38344105)	AZD4547 vs. vistusertib vs. palbociclib vs. crizotinib vs. selumetinib + docetaxel vs. AZD5363 vs. osimertinib vs. durvalumab vs. sitravatinib + AZD6738	III/IV	II	549	Recruiting	-
NCT04739358	Tepotinib	IV	I/II	65	Not yet recruiting	*MET*ex14 mutations tested by NGS, *MET* amplifications tested by FISH, *MET* fusions tested by NGS
NCT04131543 (CRETA)	Cabozantinib	IIIB/IV	II	25	Recruiting	*RET* rearrangement tested by FISH or NGS, *MET* not tested
NCT04173338 (IST-65)	Cabozantinib + pemetrexed	IIIB/IV	I	30	Recruiting	-
NCT04310007 (NCI-2020-01541)	Cabozantinib vs. cabozantinib + nivolumab vs. standard chemotherapy	III, IIIA, IIIB, IIIC, IVA, IV	II	142	Recruiting	*METex14* mutations tested by NGS, *MET* amplification tested by FISH
NCT02795156 (SCRI PRO 10)	Afatinib vs. regorafenib vs. cabozantinib	-	II	160	Recrutiung	-
NCT04514484 (NCI-2020-05956)	Cabozantinib + nivolumab	IV	I	18	Recrutiung	-
NCT03170960 (XL184-021)	Cabozantinib + atezolizumab	IV	I/II	1732	Recruiting	-
NCT04148066 (TATIN)	Osimertinib + crizotinib	IV	-	30	Recruiting	-
NCT02034981 (AcSé)	Crizotinib	IV	II	246	Active, not recruiting	One proven specific alterations among *ALK*, *MET*, *RON*, and *ROS1* genes
